# Supplementary material for: Study on Surfactant–Polymer Flooding after Polymer Flooding in High-Permeability Heterogeneous Offshore Oilfields: A Case Study of Bohai S Oilfield
Source: Polymers (Basel). 2024 Jul 12;16(14):2004. doi: 10.3390/polym16142004 (PMC11280608; doi:10.3390/polym16142004)
Supplement: Supplementary file 1 [file polymers-16-02004-s001.zip › polymers-2913972-supplementary.pdf]

Supplementary files

1.Similarity Criteria

Applying the similarity criteria, the actual reservoir conditions of S oilfield, including crude oil density, flow rate, wellbore diameter, and crude oil viscosity, were compared with the laboratory experimental conditions. By substituting these parameters into the formula, a comparison between the actual reservoir conditions of S oilfield and the laboratory reservoir conditions was conducted, as presented in Table 1.

Table S1. Comparison of Actual Reservoir Conditions in S Oilfield with Laboratory Experimental Conditions

S oilfield	Stratum / experiment	Crude oil density	Crude oil viscosity at reservoir temperature	Flow rate	Water injection rate	Hole diameter	Reynolds criterion
		($kg\cdot m^{-3}$)	（mPa·s）	($m\cdot s^{-1}$)	($m^3\cdot d^{-1}$)	(m)	/
Low viscosity core	Real formation conditions	971	45.70	0.00003	750	0.2	1.27E-04
	Laboratory experiment conditions	820	12	0.00035	0.00288	0.005	1.20E-04
Middle viscosity core	Real formation conditions	971	86.50	0.00003	800	0.2	6.74E-05
	Laboratory experiment conditions	820	21	0.00035	0.00288	0.005	6.83E-05
High viscosity core	Real formation conditions	971	291.10	0.00003	400	0.2	2.00E-05
	Laboratory experiment conditions	820	43	0.00035	0.00288	0.005	2.00E-05

2. Nuclear magnetic resonance (NMR)

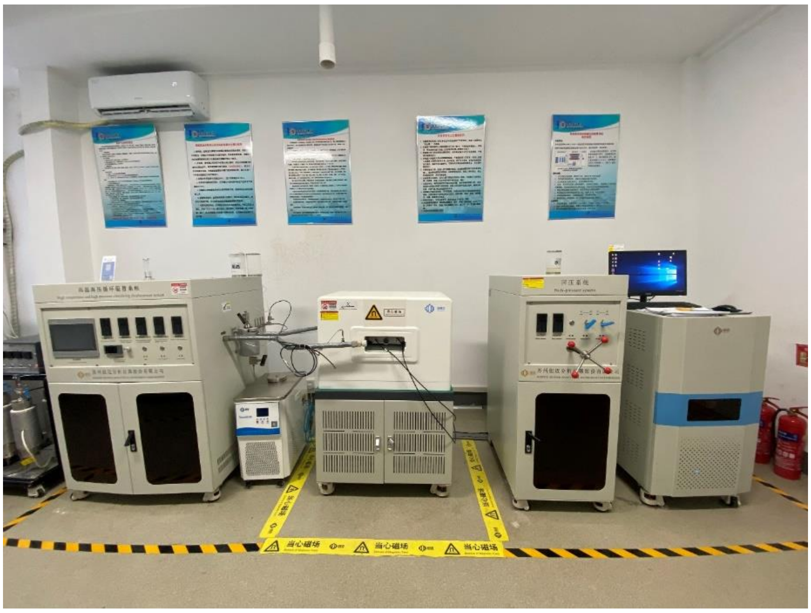

Figure S1. Newmaze Analysis High-Temperature High-Pressure Displacement Nuclear Magnetic Resonance Testing System

Analyzing the residual oil distribution and utilization patterns during the displacement process through Nuclear Magnetic Resonance (NMR) T_2 spectrum at each stage, the T_2 relaxation time reflects the chemical environment of hydrogen protons within the sample, related to the binding forces and degrees of freedom experienced by the hydrogen protons. The T_2 distribution is correlated with pore size; in a porous medium, larger pore sizes correspond to longer relaxation times for water present in the pores, while smaller pore sizes result in increased confinement and shorter relaxation times.

Measuring the NMR signals of saturated simulated reservoir water-rock cores and combining them with mercury injection capillary pressure data, it is observed from the relationship $r=\rho F_s T_2$ that with a surface relaxation rate ρ of 50 μm/s and a geometric shape factor Fs of 2 (representing a slit-shaped geometry; Fs would be 1 for a slit, 2 for a cylinder, and 3 for a sphere), there exists a certain similarity between the two, as illustrated in Figure S2.

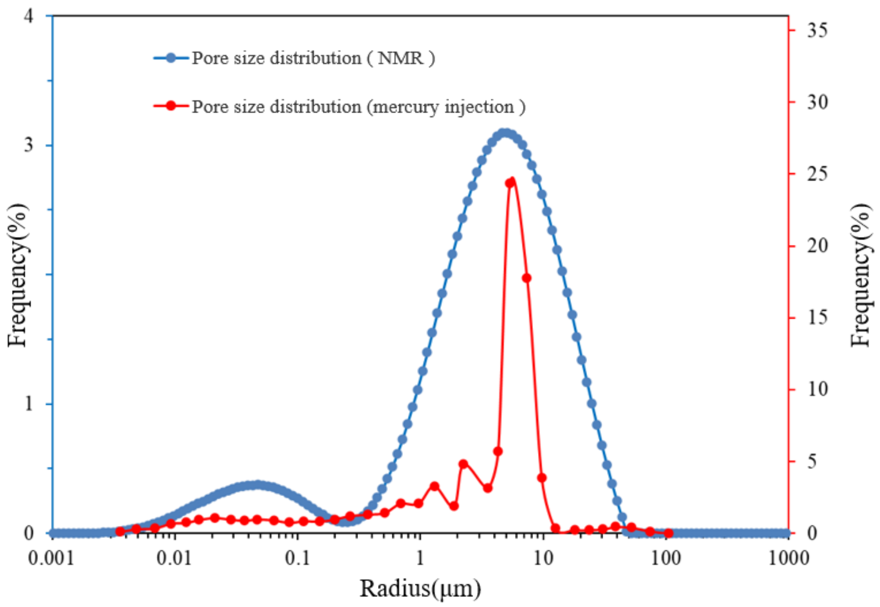

Figure S2. The conversion result of T_2 signal
